# Tibial component coverage affects tibial bone resorption and patient-reported outcome measures for patients following total knee arthroplasty

**DOI:** 10.1186/s13018-021-02250-7

**Published:** 2021-02-12

**Authors:** Changquan Liu, Guanglei Zhao, Kangming Chen, Jinyang Lyu, Jie Chen, Jingsheng Shi, Gangyong Huang, Feiyan Chen, Yibing Wei, Siqun Wang, Jun Xia

**Affiliations:** Department of Orthopedics, Huashan Hospital, Fudan University, 12, Wulumuqi Rd., Jing’an District, Shanghai, China

**Keywords:** Tibial component coverage, Under-hang, Over-hang, Alignment, Patient-reported outcome measures, Tibial bone resorption, Total knee arthroplasty

## Abstract

**Purpose:**

The aim of this study is to investigate the prognostic value of tibial component coverage (over-hang and under-hang) and the alignment of total knee arthroplasty (TKA) components 1 week after surgery. We select patient-reported outcome measures (PROMS) (the Knee Society score (KSS score) and the Western Ontario and McMaster Universities Osteoarthritis Index-pain score (WOMAC pain score)) and tibial bone resorption (TBR) 2 years after surgery as the end points.

**Methods:**

The study retrospectively analyzed 109 patients undergoing TKA (fixed-bearing prosthesis with asymmetrical tibial tray) from January 2014 to December 2017 in Huashan Hospital. By using standard long-leg X-rays, anteroposterior (AP) and lateral X-rays of the knee, tibial component coverage (under-hang or over-hang), AP tibial-femoral anatomical angle (AP-TFA), AP femoral angle (AP-FA), AP tibial angle (AP-TA), and lateral tibial angle (L-TA) were measured at 1 week after surgery, while TBR was measured through postoperative 1-week and 2-year AP and lateral radiographs of the knee on three sides (medial side, lateral side on AP radiograph, and anterior side on lateral radiograph). The Pearson correlation analysis, simple linear regression, multiple linear regression, the Student’s *t* test, and one-way ANOVA together with Tukey’s post hoc test (or Games-Howell post hoc test) were used in the analyses.

**Results:**

Tibial under-hang was more likely to appear in our patients following TKA (42%, medially, 39%, laterally, and 25%, anteriorly). In multivariate linear regression analysis of TBR, tibial under-hang (negative value) 1 week after surgery was positively correlated with TBR 2 years later on the medial (*p* = 0.003) and lateral (*p* = 0.026) side. Tibial over-hang (positive value) 1 week after surgery on the medial side was found negatively related with KSS score (*p* = 0.004) and positively related with WOMAC pain score (*p* = 0.036) 2 years later in multivariate linear regression analysis of PROMS. Both scores were better in the anatomically sized group than in the mild over-hang group (or severe over-hang) (*p* < 0.001). However, no significant relationship was found between the alignment of TKA components at 1 week after surgery and the end points (TBR and PROMS) 2 years later.

**Conclusion:**

Under-hang of the tibial component on both the medial and lateral sides can increase the risk of TBR 2 years later. Over-hang of tibial component on the medial side decreases the PROMS (KSS score and WOMAC pain score) 2 years later. An appropriate size of tibial component during TKA is extremely important for patient’s prognosis, while the alignment of components might not be as important.

**Supplementary Information:**

The online version contains supplementary material available at 10.1186/s13018-021-02250-7.

## Introduction

Total knee arthroplasty (TKA) has become the most conventional method for treating end-stage osteoarthritis of the knee, and there are studies showing that TKA is effective in the long-term follow-up [1, 2]. However, complications of TKA gradually occur over time, including aseptic loosening, infection, and pain [3–6]. To reduce the occurrence of the complications listed above and improve the functional status of patients following TKA, suitable tibial component coverage and good alignment of the components are very important [7, 8].

For tibial component coverage, it is well known that tibial component over-hang causes soft tissue irritation, postoperative pain, limited knee flexion, and poor patient-reported outcome measures (PROMS) (the Knee Injury and Osteoarthritis Outcome score (KOOS score), the Knee Society score (KSS score), and the Western Ontario and McMaster Universities Osteoarthritis Index score (WOMAC)) [9, 10]. However, some studies did not show the same results, and the effect of tibial component over-hang on postoperative PROMS is still under debate [11, 12]. Many studies have shown that the tibial component under-hang can result in the sinking of the prosthesis and increase the risk of aseptic loosening [13, 14]. Aseptic loosening is one of the late complications of TKA, and the mechanism is still not understood. Some studies have reported that tibial bone resorption (TBR) occurring within 2 years following TKA can lead to aseptic loosening [15, 16]. In a recent study, Gu et al. found that the tibial component under-hang on the medial side is positively correlated with medial TBR at 2 years after surgery [14]. It is worth mentioning that only a few studies have reported that there is a relationship between the tibial component and TBR after TKA [14, 17, 18].

For the alignment of the components, there are recommendations in the coronal and sagittal positions to obtain better results [19–21]. However, the relationship between the malalignment of components and PROMS following TKA remains controversial. Some studies have shown that the malalignment of components can cause poor PROMS [22–25], while some have shown that it does not cause poor PROMS [7, 26, 27]. Regarding aseptic loosening, many studies have shown that the malalignment of components can result in aseptic loosening in the long-term follow-up, but only a few studies have reported the relationship between the alignment of components and TBR in the short-term follow-up [14, 28, 29].

The objective of the present study is to determine whether there are any relationships between these variables (tibial component coverage and TKA component alignment) 1 week after surgery and the end points (PROMS (KSS score, WOMAC pain score) and TBR) at 2 years after surgery.

## Methods

### Patient selection

**Fig. 1:**
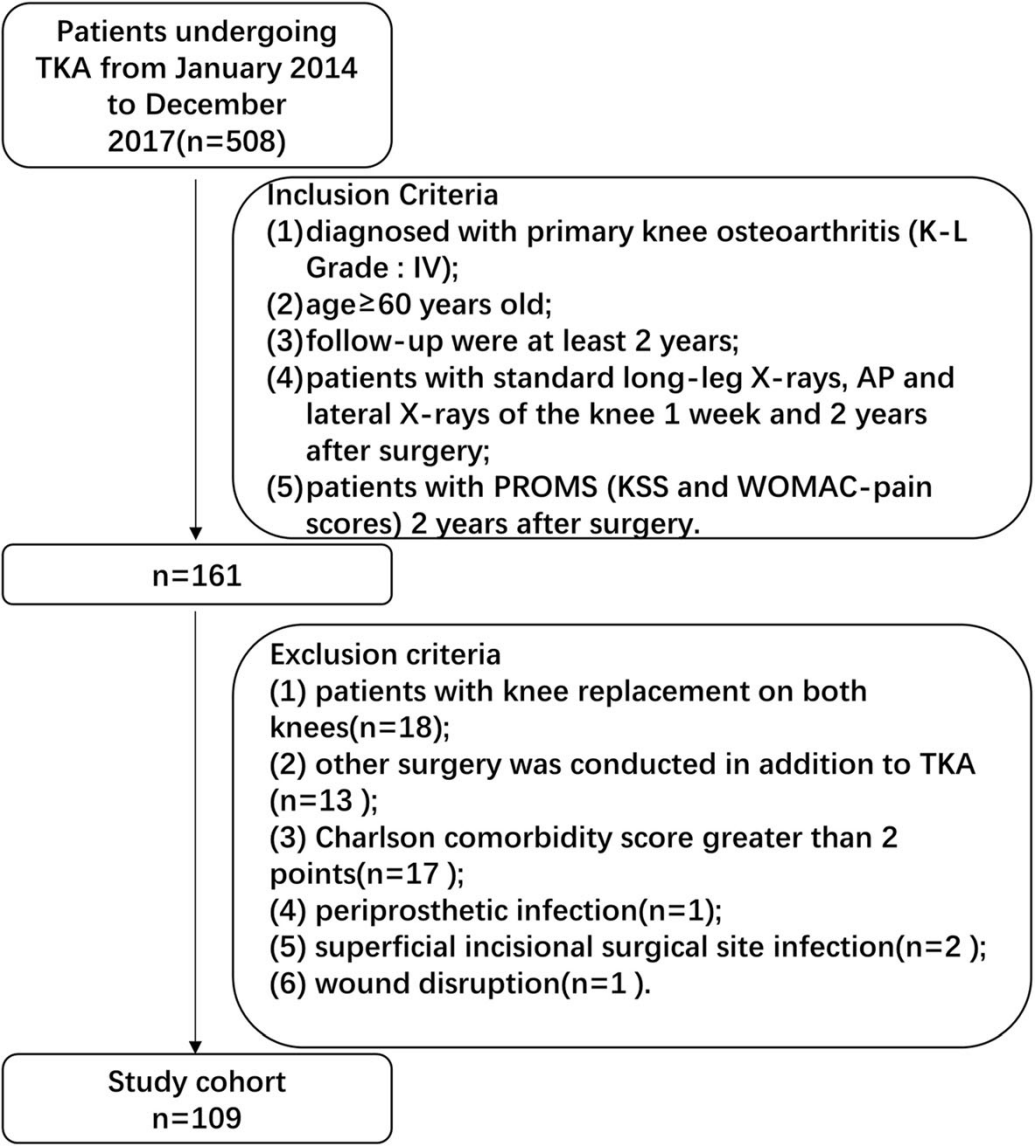
Flow chart

This study retrospectively analyzed patients undergoing TKA from January 2014 to December 2017 in the Department of Orthopedics at Huashan Hospital of Fudan University (*n* = 508). The knee system (LEGION™, Smith & Nephew) used in our center was a fixed-bearing prosthesis with an asymmetrical tibial tray. The medium-viscosity bone cement VERSABOND (Smith & Nephew, USA) was used to fix the tibial component. All surgeries were performed under a tourniquet by two senior joint surgeons. The inclusion criteria were as follows: (1) a diagnosis of primary knee osteoarthritis (Kellgren & Lawrence Grade: IV); (2) age ≥ 60 years old; (3) follow-up data for at least 2 years; (4) patients with standard long-leg X-rays, anteroposterior (AP) and lateral X-rays of the knee at 1 week and 2 years after surgery; and (5) patients with PROMS (KSS and WOMAC pain score) at 2 years after surgery. The exclusion criteria were as follows: (1) patients with knee replacement in both knees (*n* = 18); (2) other surgery was conducted in addition to TKA (*n* = 13); (3) Charlson comorbidity score greater than 2 points (*n* = 17); (4) periprosthetic infection (*n* = 1); (5) superficial incisional surgical site infection (*n* = 2); and (6) wound disruption (*n* = 1). Finally, we included a total of 109 cases for analysis (Fig. 1). The basic characteristics of all patients are shown in Table 1. The study was approved by the ethics committee of Huashan Hospital of Fudan University.

**Table 1:** Basic characteristics

Variables	Continuous variables: mean ± SD (range); categorical variables: frequency (%)
**Age (years)**	68.12 ± 5.39 (60.00 to 86.00)
**Height (m)**	159.78 ± 6.52 (141.00 to 182.00)
**Weight (kg)**	67.23 ± 9.03 (42.00 to 90.00)
**BMI (kg/m2)**	26.34 ± 3.33 (17.48 to 36.22)
**Sex**	
Male	39 (36%)
Female	70 (64%)
**Side**	
Right	49.00 (45%)
Left	60.00 (55%)
**Tibial component coverage (mm)**	
Medial	−0.98 ± 2.37 (−7.39 to 5.29)
Lateral	−0.69 ± 2.00 (−5.44 to 5.63)
Anterior	−0.06 ± 1.63 (− 5.98 to 3.62)
**Tibial bone resorption (mm)**	
Medial	2.00 ± 2.38 (−2.25 to 9.24)
Lateral	1.04 ± 2.52 (−3.14 to 9.37)
Anterior	0.89 ± 1.93 (− 3.83 to 6.95)
**AP-FA (°)**	95.04 ± 2.05 (90.80 to 99.20)
**AP-TA (°)**	89.65 ± 2.30 (84.20 to 95.40)
**L-TA (°)**	86.48 ± 1.77 (81.50 to 90.80)
**AP-FTA (°)**	184.69 ± 3.03 (177.50 to 192.60)
**KSS score**	
KSS knee-pre	47.20 ± 10.22 (23.00 to 70.00)
KSS function-pre	54.08 ± 12.06 (25.00 to 75.00)
KSS total-pre	101.28 ± 21.78 (49.00 to 140.00)
KSS knee-post	88.28 ± 6.63 (62.00 to 98.00)
KSS function-post	89.31 ± 7.89 (65.00 to 100.00)
KSS total-post	177.60 ± 13.81 (127.00 to 197.00)
**WOMAC pain score**	
WOMACp-pre	17.24 ± 1.86 (13.00 to 20.00)
WOMACp-post	3.04 ± 2.09 (0.00 to 8.00)

*SD* standard deviation; *BMI* body mass index; tibial component coverage, over-hang: positive value, under-hang: negative value; *AP-FA* anteroposterior femoral angle; *AP-TA* anteroposterior tibial angle; *L-TA* lateral tibial angle; *AP-FTA* anteroposterior tibial-femoral anatomical angle; *KSS score*, the Knee Society score; *WOMAC score* the Western Ontario and McMaster Universities Osteoarthritis Index score

### Patient-reported outcome measures

In this study, two PROMS were used preoperatively and postoperatively (2 years after surgery), including the Knee Society score (KSS) and the Western Ontario and McMaster Universities Osteoarthritis index (WOMAC). The KSS score, which is a questionnaire designed to evaluate the knee of patients, includes two parts: a knee score and a function score, both of which score from 0 to 100, with higher scores representing a better status [30]. The WOMAC score, which is a self-administered questionnaire used to assess the osteoarthritic hip and knee of patients, consists of three parts: a pain subscale, stiffness subscale, and function subscale. Only the WOMAC pain subscale (scores from 0 to 20, high scores represent worse status) was used in our study to evaluate the pain status of patients [31].

### Radiographic measurement

All patients had standard long-leg X-rays, AP, and lateral X-rays of the knee at 1 week (routine examination after surgery) and 2 years after surgery. The radiographs were analyzed and measured through the hospital’s imaging system (GE Medical Systems) by two experienced orthopedists. The two orthopedists were not involve in the surgery.

**Fig. 2:**
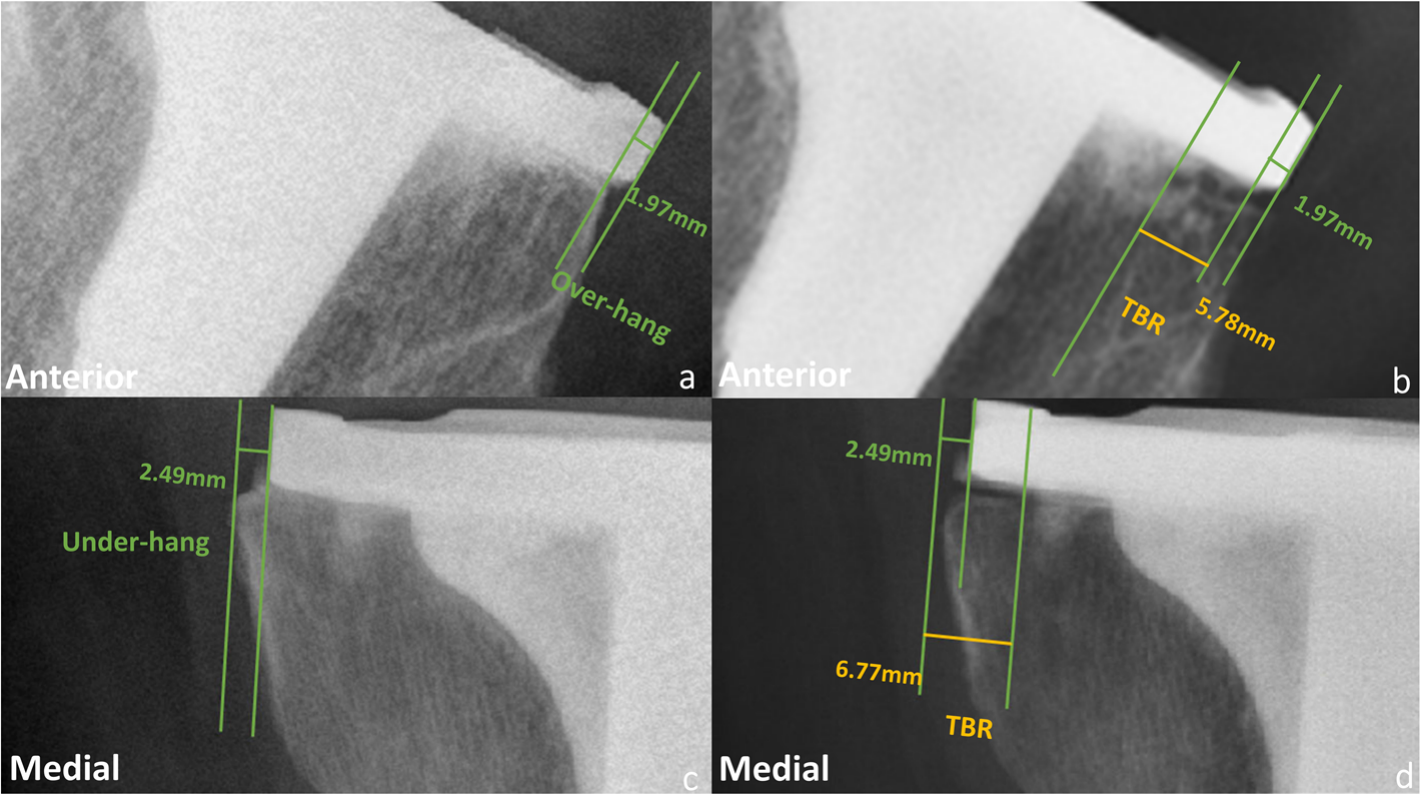
A pattern diagram showing tibial component over-hang (**a**), under-hang (**c**) at 1 week after surgery, and tibial bone resorption (TBR) (**b**, **d**) at 2 years after surgery on both AP (**a**, **b**) and lateral (**c**, **d**) radiographs. Tibial component over-hang (positive value) or under-hang (negative value) is defined as the distance between the tangent line of the tibial baseplate and the edge of the ipsilateral tibial cutting surface 1 week after surgery. Tibial bone resorption (TBR) is defined as the distance between the edge of the tibial cutting surface (1 week after surgery) and the closure of bone resorption (2 years after surgery). The distance greater than 1 mm was considered as a valid TBR

On the AP radiograph taken 1 week after surgery, the distance between the tangent line of the tibial baseplate and the edge of the ipsilateral tibial cutting surface was measured to determine whether the tibial component exhibited over-hang (positive value) or under-hang (negative value) [14]. On the lateral radiograph taken 1 week after surgery, the measurements of over-hang and under-hang were the same (Fig. 2). According to previous studies, over-hang could be classified into three groups: anatomically sized, 0 mm ≤ distance < 1 mm; mild over-hang, 1 mm ≤ distance < 3 mm; severe over-hang, distance ≥3 mm [11, 12].

TBR was defined as the distance between the edge of the tibial cutting surface and the closure of bone resorption, which was measured through postoperative 1-week and 2-year AP and lateral radiographs on three sides (medial side, lateral side on the AP radiograph, and anterior side on the lateral radiograph) (Fig. 2). The posterior side on the lateral radiograph was excluded because slight rotation of the lateral radiograph can cause overlap of the posterior condyle, which prevents the position of the tibial component from being assessed accurately. A distance greater than 1 mm was considered to indicate valid TBR. Positive values represented the progress of bone resorption, while negative values represented the formation of new bone after 2 years [14]. Good inter-observer reliability of the TBR measurements was shown between the two observers using the intraclass correlation coefficient (ICC) (ICC medially = 0.996, ICC laterally = 0.998, ICC anteriorly = 0.994).

**Fig. 3:**
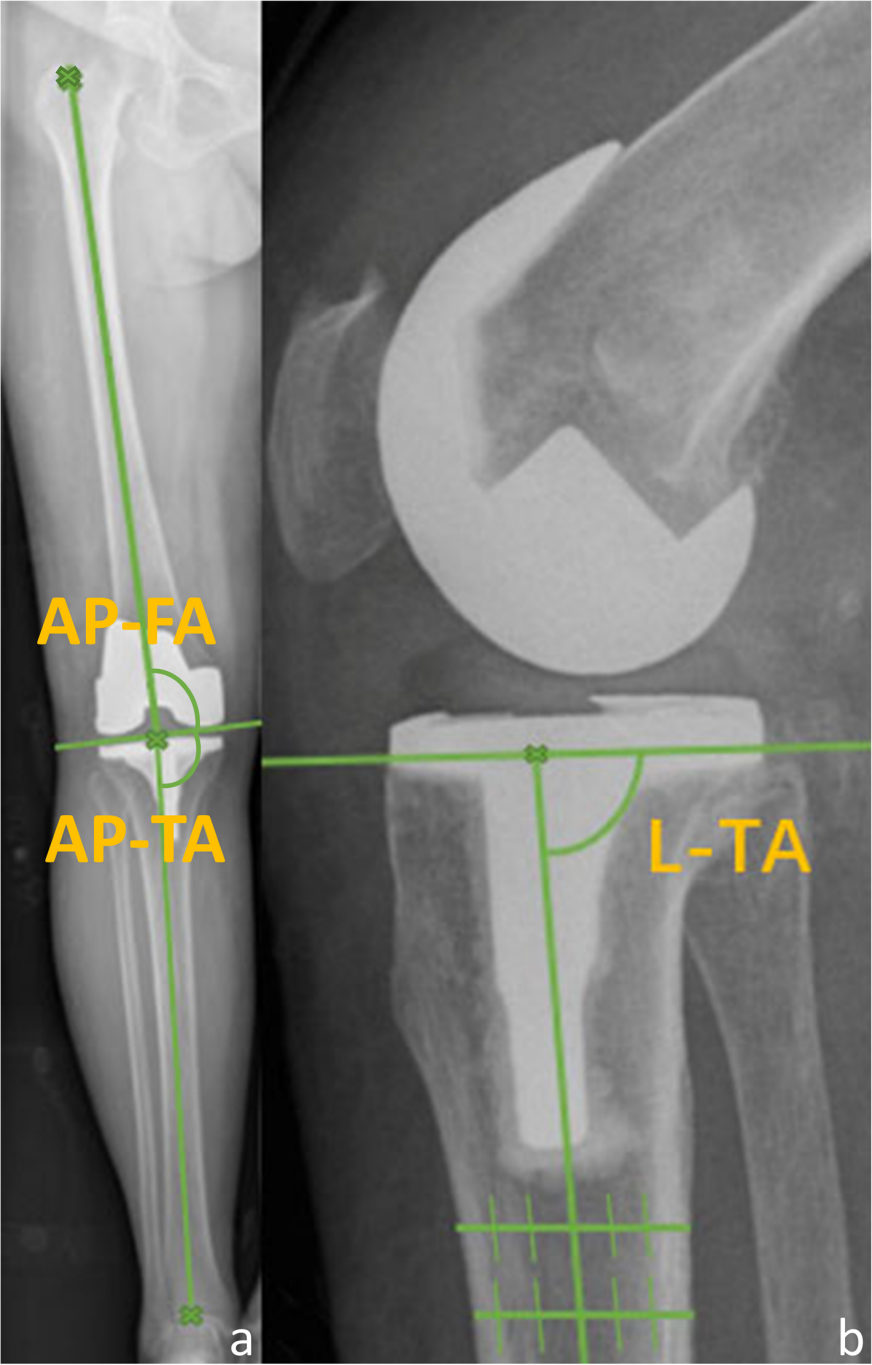
A pattern diagram of different alignment parameters on long-leg radiographs (**a**) and lateral radiographs of the knee (**b**) at 1 week after surgery. AP-FA, AP femoral angle, which is the angle between the femoral component axis and the coronal anatomical axis of femoral shaft; AP-TA, AP tibial angle, which is the angle between the tibial plate and the coronal anatomical axis of tibial shaft; L-TA, lateral tibial angle, which is the angle between the tibial plate and the sagittal anatomical axis of tibial shaft; AP-TFA, AP tibial-femoral anatomical angle, which is the angle combining AP-FA and AP-TA

The long-leg X-rays and lateral radiographs of the knee taken 1 week after surgery were used to measure different alignment parameters of the TKA components. AP tibial-femoral anatomical angle (AP-TFA), AP femoral angle (AP-FA), AP tibial angle (AP-TA), and lateral tibial angle(L-TA) were measured. AP-FA is the angle between the femoral component axis and the coronal anatomical axis of the femoral shaft, AP-TA is the angle between the tibial plate and the coronal anatomical axis of the tibial shaft and the AP-TFA is the angle combining AP-FA and AP-TA. L-TA is the angle between the tibial plate and the sagittal anatomical axis of the tibial shaft [12, 32] (Fig. 3 and Table 2).

**Table 2:** Classification of alignment

	Long-leg X-rays				Lateral X-rays of the knee	
	Aligned	Varus	Valgus		Aligned	Misaligned
AP-FA	92° to 98°	< 92°	> 98°			
AP-TA	87° to 93°	< 87°	> 93°	L-TA	83° to 90°	< 83° or > 90°
AP-TFA	183° to 187.5°	< 183°	> 187.5°			

*AP-FA* anteroposterior femoral angle; *AP-TA* anteroposterior tibial angle; *AP-FTA* anteroposterior tibial-femoral anatomical angle; *L-TA* lateral tibial angle

### Sample size calculation

We chose TBR 2 at years after TKA as the main outcome indicator for the sample size calculation. In a recent study, Martin et al. studied the influence of different tibial tray thicknesses on TBR at 2 years after surgery. The authors found that the mean medial TBR (1.1 ± 1.3 mm) with the thick tibial tray was significantly larger than that with the thin tibial tray (0.2 ± 0.5 mm) [16]. TBR was considered clinically significant only when the width was approximately 1 mm, as a width of ≤ 1 mm can be the result of insufficient cement penetration [14]. The power analysis conducted using G*Power, version 3.1.9.7, showed that *n* = 50 was the minimum sample size required, with an alpha error of 0.05 and power of 90%. We included a total of 109 patients in this study.

### Statistical analysis

The continuous variables were presented as means ± standard deviations (SD) (range) and the categorical variables were presented as frequencies with percentages (%). The Pearson correlation analysis was performed between tibial component coverage and the end points (TBR and PROMS (KSS total-post and WOMAC pain-post)). Simple linear regression was carried out between the independent variables (age, BMI, sex, side, tibial component coverage, KSS total-pre, and WOMAC pain-pre) and dependent variables (TBR and PROMS (KSS total-post and WOMAC pain-post)). The variables (*p* < 0.10 in simple linear regression) were further analyzed in multiple linear regression. Student’s *t* test and one-way ANOVA together with Tukey’s post hoc test (or Games-Howell post hoc test) were used to test the association between component variables (alignment and over-hang) and the end points (TBR and PROMS (KSS total-post and WOMAC pain-post)). All analyses were performed using SPSS 24.0, and *p* < 0.05 (two-tailed) was regarded statistically significant.

## Results

**Fig. 4:**
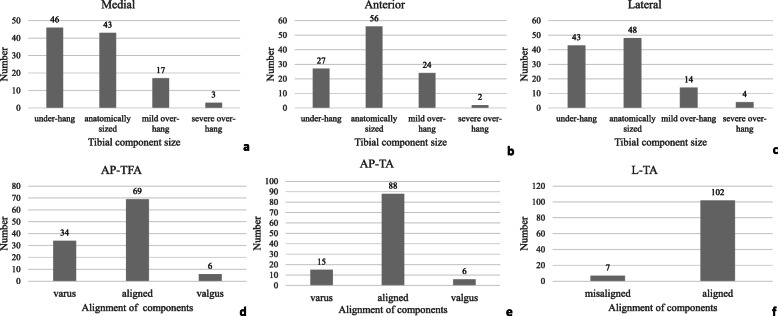
Distribution of tibial component size (medial, lateral, and anterior side) (**a**, **b**, **c**) and different alignment parameters (AP-TFA, AP-TA, and L-TA) (**d**, **e**, **f**) at 1 week after surgery. Tibial component sizes are classified into three groups: anatomically sized, 0 mm ≤ distance < 1 mm; under-hang, distance ≥ 1 mm; over-hang: mild over-hang, 1 mm ≤ distance < 3 mm, severe over-hang, distance ≥ 3 mm. AP-TFA, anteroposterior (AP) tibial-femoral anatomical angle, aligned: 183° ≤ angle ≤ 187.5°, varus: angle < 183°, valgus: angle > 187.5°; AP-TA, AP tibial angle, aligned: 87° ≤ angle ≤ 93°, varus: angle < 87°, valgus: angle > 93°; L-TA, lateral tibial angle, aligned: 83° ≤ angle ≤90°, misaligned: angle < 83°and angle > 90°.

The mean ± SD (range) of age and BMI were 68.12 ± 5.39 (60.00 to 86.00) and 26.34 ± 3.33(17.48 to 36.22), respectively. Of all patients, 70 patients were female, while only 39 patients were male; 49 patients underwent TKA surgery on the right knee, and the others (60 patients) underwent TKA surgery on the left knee (Table 1). The mean (± SD) and range of tibial component coverage (medial, lateral, and anterior side), TBR (medial, lateral, and anterior side), AP-FA, AP-TA, L-TA, AP-FTA, KSS score (KSS knee-pre, KSS function-pre, KSS total-pre, KSS knee-post, KSS function-post, and KSS total-post), WOMAC pain score (WOMAC pain-pre and WOMAC pain-post) are presented in Table 1. Tibial component coverage of the patients was more likely to have an under-hang status in our center. Under-hang was present in 42%, 39%, and 25% of the cases on the medial, lateral, and anterior sides, respectively, while over-hang was present in 18%, 17%, and 24% of cases on the medial, lateral, and anterior sides, respectively (Fig. 4a, b, c). For the integrated alignment of TKA components (AP-TFA), most of the patients exhibited varus (31%) or aligned (63%), but there were still a few (6%) cases of minor valgus (Fig. 4d).

In Pearson correlation analysis, tibial under-hang (negative value) at 1 week after surgery was positively related to TBR at 2 years after surgery on the medial (*r* = − 0.30, *p* = 0.001) and lateral (*r* = − 0.21, *p* = 0.029) sides of the tibial component. There were also correlations between tibial over-hang (positive value) at 1 week after surgery and PROMS (KSS total-post, *r* = − 0.28, *p* = 0.003, negatively; WOMAC pain-post, *r* = 0.21, *p* = 0.026, positively) at 2 years after surgery (Table 3).

**Table 3:** Pearson correlation analysis between tibial component coverage at 1 week after surgery and tibial bone resorption, patient-reported outcome measures (PROMS) (KSS total-post and WOMAC pain-post) at 2 years after surgery

	Tibial component coverage (mm)	Tibial bone resorption (mm)				Patient-reported outcome measures			
	Mean ± SD (range)	Medial	Lateral	Anterior	*p*	KSS total-post	***p***	WOMAC pain-post	***p***
**Medial**	−0.98 ± 2.37 (−7.39 to5.29)	−0.30			**0.001**	− 0.28	**0.003**	0.21	**0.026**
**Lateral**	−0.69 ± 2.00 (−5.44 to5.63)		−0.21		**0.029**	0.07	0.467	−0.09	0.366
**Anterior**	−0.06 ± 1.63 (− 5.98 to3.62)			−0.17	0.076	0.08	0.403	0.00	0.983

Tibial component coverage, over-hang: positive value, under-hang: negative value; *SD* standard deviation; *KSS score* the Knee Society score; *WOMAC score* the Western Ontario and McMaster Universities Osteoarthritis Index score

In simple linear regression analysis of TBR at 2 years after surgery, only tibial under-hang (negative value, *p* = 0.001, medially; *p* = 0.029, laterally) at 1 week after surgery was positively correlated with TBR 2 years later on the medial and lateral sides of the tibial component (Table 4). In further multiple linear regression analysis, tibial under-hang (negative value, *p* = 0.003, medially; *p* = 0.026, laterally) at 1 week after surgery was still positively correlated with TBR at 2 years after surgery on the medial and lateral sides (Table 6).

**Table 4:** Simple linear regression of tibial bone resorption at 2 years after surgery

Variables	Simple linear regression								
	Tibial bone resorption (mm)								
	Medial			Lateral			Anterior		
	*β*	95% CI	*p*	*β*	95% CI	*p*	*β*	95% CI	*p*
**Age (years)**	0.004	−0.081 to 0.088	0.934	0.008	−0.082 to 0.097	0.866	0.053	−0.015 to 0.120	0.127
**BMI (kg/m**^**2**^**)**	0.135	0.000 to 0.269	**0.050**	−0.030	−0.175 to 0.115	0.685	−0.045	−0.156 to 0.065	0.418
**Sex**	−0.246	−1.197 to 0.706	0.610	−0.380	−1.385 to0.625	0.455	−0.252	−1.021 to 0.518	0.518
**Side**	0.043	−0.875 to 0.961	0.926	0.411	−0.557 to1.379	0.402	0.149	−0.593 to 0.891	0.691
**Tibial component coverage (mm)**									
Medial	−0.303	−0.486 to − 0.119	**0.001**						
Lateral				−0.263	−0.499 to −0.027	**0.029**			
Anterior							−0.202	−0.424 to 0.021	**0.076**
**KSS score**									
KSS total-pre	0.000	−0.021 to 0.021	0.990	−0.007	−0.029 to 0.016	0.557	0.004	−0.013 to 0.021	0.670
**WOMAC pain score**									
WOMACp-pre	0.071	−0.174 to 0.316	0.567	0.149	−0.109 to 0.407	0.255	0.005	−0.193 to 0.204	0.957

*CI* confidence interval; *BMI*, body mass index; tibial component coverage, over-hang: positive value, under-hang: negative value; *KSS score* the Knee Society score; *WOMAC score*, the Western Ontario and McMaster Universities Osteoarthritis index score

In simple linear regression analysis of PROMS (KSS score and WOMAC pain score), only tibial over-hang (positive value) at 1 week after surgery on the medial side (*p* = 0.003, negatively) and KSS total-pre (*p* = < 0.001, positively) were found to have a relationship with KSS total-post 2 years later. The same two variables (*p* = 0.026, medial tibial over-hang (positive value), positively; *p* = 0.008, KSS total-pre, negatively) showing correlation with WOMAC pain-post 2 years later (Table 5). In further multiple linear regression analysis, tibial over-hang (positive value) at 1 week after surgery on the medial side (*p* = 0.004, negatively) and KSS total-pre-score (*p* = < 0.001, positively) were still correlated with the KSS score, as well as with the WOMAC pain score 2 years later (*p* = 0.036, medial tibial over-hang (positive value), positively; *p* = 0.011, KSS total-pre, negatively) (Table 7).

**Table 5:** Simple linear regression of patient-reported outcome measures PROMS at 2 years after surgery (KSS total-post and WOMAC pain-post)

Variables	Simple linear regression					
	KSS total-post			WOMAC pain-post		
	*β*	95% CI	*p*	*β*	95% CI	*p*
**Age (years)**	−0.237	−0.726 to 0.252	0.339	0.055	−0.019 to 0.128	0.144
**BMI (kg/m**^**2**^**)**	0.223	−0.571 to 1.018	0.579	−0.066	−0.185 to 0.054	0.277
**Sex**	0.908	−4.609 to 6.425	0.745	−0.337	−1.168 to 0.494	0.424
**Side**	−0.919	−6.235 to 4.398	0.733	−0.341	−1.142 to 0.459	0.400
**Tibial component coverage (mm)**						
Medial	−1.652	−2.723 to −0.581	**0.003**	0.187	0.022 to 0.352	**0.026**
Lateral	0.485	−0.831 to 1.802	0.467	−0.091	−0.289 to 0.108	0.366
Anterior	0.683	−0.931 to 2.298	0.403	0.003	−0.242 to 0.247	0.983
**KSS score**						
KSS total-pre	0.244	0.132 to 0.356	**< 0.001**	−0.024	−0.042 to −0.006	**0.008**
**WOMAC pain score**						
WOMACp-pre	−0.679	−2.094 to 0.736	0.344	−0.008	−0.222 to 0.207	0.943

*CI* confidence interval; *BMI* body mass index; tibial component coverage, over-hang: positive value, under-hang: negative value; *KSS score*, the Knee Society Score; *WOMAC score*, the Western Ontario and McMaster Universities Osteoarthritis index score

Regarding the relationship between over-hang (anatomically sized, mild over-hang, and severe over-hang) and the two end points (TBR and PROMS), TBR (including the medial, lateral, and anterior side) did not differ significantly across the categories of over-hang (including anatomically sized vs mild over-hang, anatomically sized vs severe over-hang and mild over-hang vs severe over-hang) (Table S1). PROMS (KSS total-post and WOMAC pain-post) differed significantly across the categories of over-hang on the medial side, and both scores were better in the anatomically sized group than in the mild over-hang group (or severe over-hang) (*p* < 0.001) (Table 8).

There was no significant difference in TBR (including the medial, lateral, and anterior side) 2 years later across the types of alignment of the TKA component (AP-TA, L-TA, AP-FTA) at 1 week after surgery (Table S2) or in the PROMS (KSS total-post and WOMAC pain-post) (Table S3).

## Discussion

In this study, the important findings were that tibial under-hang at 1 week after surgery can promote TBR 2 years later on the medial and lateral sides, and tibial over-hang can decrease KSS score and WOMAC pain score for the medial side recorded 2 years later. However, the alignment of the TKA components at 1 week after surgery did not have a significant relationship with either end point.

Some studies have focused on aseptic loosening or TBR in patients undergoing TKA [14, 16, 29]. Regarding the relationship between the tibial component size and TBR, to date, only one study has reported TBR at 2 years following TKA due to under-hang of the tibial component [14]. We found similar results in our research (Table 3 and Table 6). These results indicated that under-hang of the tibial component might increase the probability of aseptic loosening and result in prosthesis failure, so orthopedists should prevent under-hang on the medial and lateral sides during the TKA procedure. However, to avoid over-hang and malrotation of the tibial components, surgeons often choose a smaller prosthesis when over-hang is found during surgery, which often leads to the occurrence of under-hang [33, 34]. This can also explain our finding shown in Fig. 4. It makes sense that orthopedists installed proper tibial component on the medial and lateral sides during the process of TKA instead of under-hang status.

**Table 6:** Multiple linear regression of tibial bone resorption at 2 years after surgery

Variables	Multiple linear regression								
	Tibial bone resorption (mm)								
	Medial			Lateral			Anterior		
	*β*	95% CI	*p*	*β*	95% CI	*p*	*β*	95% CI	*p*
**BMI (kg/m**^**2**^**)**	0.127	0.002 to 0.256	**0.054**	−0.008	−0.152 to 0.137	0.917	−0.039	−0.149 to 0.071	0.489
**Tibial component coverage (mm)**									
Medial	−0.296	−0.478 to −0.115	**0.002**						
Lateral				−0.261	−0.500 to −0.022	**0.032**			
Anterior							−0.196	−0.420 to 0.028	0.086

*CI* confidence interval; *BMI* body mass index; tibial component coverage, over-hang: positive value, under-hang: negative value

The effect of tibial component coverage on postoperative PROMS has long been debated [8–12, 35]. Nielsen et al. [8], Bonnin et al. [9, 35], and Simsek et al. [10] investigated that over-hang can negatively affect PROMS after surgery. However, Ahmed et al. [12] and Abram et al. [11] found that tibial over-hang does not influence PROMS after TKA. Our findings were consistent with the former results. In our study, we recorded the exact location of the over-hang and found that tibial over-hang on the medial side can negatively influence the PROMS (KSS score WOMAC pain score) 2 years after TKA (Tables 3, 7, and 8). It is worth mentioning that Nielsen et al. [8] also investigated that tibial over-hang medially was correlated with poor outcome (KOOS pain < 70) in a prospective cohort of 323 patients. These findings might be explained by the fact that the medial collateral ligament was on the medial side of the knee and was prone to irritation due to over-hang of the tibial component. This, to some extent, reminds doctors to avoid medial over-hang of the tibial component, even if over-hang is inevitable during surgery.

**Table 7:** Multiple linear regression of patient-reported outcome measures PROMS (KSS total-post and WOMAC pain-post) at 2 years after surgery

Variables	Multiple linear regression					
	KSS total-post			WOMAC pain-post		
	*β*	95% CI	*p*	*β*	95% CI	*p*
**Tibial component coverage (mm)**						
Medial	−1.502	−2.500 to −0.505	**0.004**	0.172	0.011 to 0.334	**0.036**
Lateral						
Anterior						
**KSS score**						
KSS total-pre	0.233	0.124 to 0.341	**< 0.001**	−0.023	−0.040 to −0.005	**0.011**

*CI* confidence interval; tibial component coverage, over-hang: positive value, under-hang: negative value; *KSS score*, the Knee Society score; *WOMAC score*, the Western Ontario and McMaster Universities Osteoarthritis index score

**Table 8:** Association between over-hang and PROMS (KSS total-post and WOMAC pain-post) at 2 years after surgery

	Anatomically sized^**1**^	Mild over-hang^**1**^	Severe over-hang^**1**^	***P***^**2**^	***P***^**3**^ (mild vs anatomically sized)	***P***^**3**^ (sever vs anatomically sized)	***P***^**3**^ (severe vs mild)
	Mean ± SD (range)						
**Association between medial over-hang and PROMS 2 years after surgery**							
**KSS total-post**	183.93 ± 10.92 (127 to 196)	161.12 ± 8.37 (149 to 174)	150.67 ± 16.50 (137 to 169)	**< 0.001**	**< 0.001**	**< 0.001**	0.555
**WOMAC pain-post**	2.07 ± 1.55 (0 to 6)	5.29 ± 1.65 (3 to 8)	6.33 ± 2.08 (4 to 8)	**< 0.001**	**< 0.001**	**< 0.001**	0.261
**Association between lateral over-hang and PROMS 2 years after surgery**							
**KSS total-post**	178.33 ± 14.82 (127 to 196)	177.71 ± 15.52 (149 to 197)	185.75 ± 7.18 (175 to 190)	0.605	0.989	0.599	0.602
**WOMAC pain-post**	2.77 ± 2.16 (0 to 8)	2.79 ± 2.22 (0 to 7)	2.25 ± 1.50 (1 to 4)	0.894	1.000	0.887	0.899
**Association between anterior over-hang and PROMS 2 years after surgery**							
**KSS total-post**	177.86 ± 12.31 (127 to 194)	177.54 ± 13.80 (150 to 197)	192.50 ± 0.71 (192 to 193)	0.272	0.994	0.250	0.251
**WOMAC pain-post**	2.91 ± 1.90 (0 to 8)	3.42 ± 2.04 (0 to 8)	1.00 ± 1.41 (0 to 2)	0.193	0.535	0.361	0.213

*SD* standard deviation; *PROMS* patient-reported outcome measures; *KSS score* the Knee Society score; *WOMAC score*, the Western Ontario and McMaster Universities Osteoarthritis index score

^1^Anatomically sized, 0 mm-1 mm; mild over-hang, 1 mm-3 mm; severe over-hang, ≥ 3 mm

^2^One-way ANOVA

^3^Tukey’s post hoc test or Games-Howell post hoc test

The relationship between the alignment and the PROMS after surgery remains unclear [7, 22–27, 36]. Rassir et al. [22] and William et al. [36] discovered that the alignment had a large influence on PROMS postoperatively. While Slevin et al. [26] and Rames et al. [27] did not investigate the same results. In our research, no correlations were found between the alignment of components at 1 week after surgery and PROMS at 2 years after surgery, which is consistent with the latter results (Table S3). It is worth mentioning that in recent years, an increasing number of orthopedists have advocated for kinematic alignment, which aims to restore preoperative anatomical alignment, often leading to mild varus [2, 37, 38]. In a cohort of 217 patients followed up for 10 years, Howell et al. [2] investigated that there was no discrepancy in PROMS (OKS and WOMAC) among different alignments in cases kinematically aligned during TKA. In our research, varus (31%%) and aligned (63%) coronal anatomical alignments were the majority (94%) (Fig. 4), which might explain why we did not find any relationships between alignment and PROMS in our research. For TBR at 2 years after surgery, no correlations were found with the alignment of components at 1 week after surgery in our research (Table S2). Some studies have assessed the correlation between the alignment of components and tibial component loosening or migration [29, 39–41]. Our result was similar with that in a previous study, in which Abdel et al. [41] discovered that the incidence of tibial loosening did not significantly differ according to coronal alignment.

### Limitations

Our research has its limitations. First, our research was a retrospective study in one orthopedic center, and we included a relatively small number of patients based on the inclusion and exclusion criteria. A prospective study with more cases should be conducted to confirm the results. Second, malrotation of the components might have an unexpected influence on the extent of coverage (under-hang or over-hang) and alignment status. It is better to study the relationship between the malrotation of components and the coverage status, as well as the influence of component malrotation on TBR and PROMS at 2 years after surgery. However, a CT scan is needed to measure the degree of rotation of components and only a few patients included in our analysis underwent CT scans. Third, the X-rays were not strictly standardized in some cases, while the rotation of the tibia might change the amount of TBR on the radiograph. Fourth, as no power calculation was performed for the sample size needed to assess the effect of alignment in our study, any lack of difference is potentially a type II error (underpowered). Last, after a follow-up period of 2 years, only TBR, not aseptic loosening, occurred in our cohort. Studies with a longer follow-up are needed to detect the relationship between TBR at 2 years after surgery and long-term complication-aseptic loosening.

## Conclusion

Under-hang of the tibial component on both the medial and lateral sides can increase the risk of TBR 2 years later. Over-hang of tibial component on the medial side decreases the PROMS (KSS score and WOMAC pain score) 2 years later. Neither TBR nor PROMS 2 years later were found to be significantly related to alignment of components. An appropriate size of the tibial component during TKA is extremely important for patient’s prognosis, while the alignment of components might not be as important.

## Supplementary Information

**Additional file 1: **Table S1. Association between over-hang and tibial bone resorption at 2 years. **Table S2**. Association between alignment and tibial bone resorption at 2 years after surgery. **Table S3**. Association between alignment and PROMS (KSS total-post and WOMAC pain-post) at 2 years after surgery

## Data Availability

All the data are available in contact with the corresponding author.

## Abbreviations

AP: Anteroposterior
AP-FA: AP femoral angle
AP-TA: AP tibial angle
AP-TFA: AP tibial-femoral anatomical angle
BMI: Body mass index
CI: Confidence interval
CT: Computed tomography
HKA: Hip-knee-ankle
KSS: The Knee Society score
KOOS: The Knee Injury and Osteoarthritis Outcome score
L-TA: Lateral tibial angle
OKS: Oxford Knee score
PROMS: Patient-reported outcome measures
SD: Standard deviations
SF-12: Short Form-12
TBR: Tibial bone resorption
TKA: Total knee arthroplasty
WOMAC: Western Ontario and McMaster Universities Osteoarthritis Index score
