# Supplementary material for: Tibial component coverage affects tibial bone resorption and patient-reported outcome measures for patients following total knee arthroplasty
Source: J Orthop Surg Res. 2021 Feb 12;16:134. doi: 10.1186/s13018-021-02250-7 (PMC7881541; doi:10.1186/s13018-021-02250-7)
Supplement: Supplementary file 1 — Additional file 1: Table S1. Association between over-hang and tibial bone resorption at 2 years. Table S2. Association between alignment and tibial bone resorption at 2 years after surgery. Table S3. Association between alignment and PROMS (KSS total-post and WOMAC pain-post) at 2 years after surgery [file 13018_2021_2250_MOESM1_ESM.docx]

**Table S1. Association between over-hang and tibial bone resorption at 2 years after surgery.**

|  | **Anatomically sized^1^** | **Mild over-hang^1^** | **Sever over-hang^1^** |  | **P^2^** | **P^3^** | **P^3^** | **P^3^** |
| --- | --- | --- | --- | --- | --- | --- | --- | --- |
|  |  | | |  |  | (mild VS Anatomically sized) | (sever VS anatomically sized) | (sever VS  mild) |
|  | **Mean ±SD (range)** | | |  |  |  |  |  |
|  |  | | |  |  |  |  |  |
| **Association between medial overhang and medial-TBR 2 years after surgery** | | | | | | | | |
| **Medial-TBR** | 1.61±1.95  (-1.67 to 8.21) | 1.18±2.07  (-1.56 to 5.89) | 0.93±0.86  (0.17 to 1.86) |  | 0.668 | 0.729 | 0.830 | 0.976 |
| **Association between lateral overhang and lateral-TBR 2 years after surgery** | | | | | | | | |
| **Lateral-TBR** | 1.21±2.41  (-2.93 to 7.43) | -0.38±1.35  (-2.20 to 2.15) | -1.38±1.99  (-2.69 to 1.57) |  | 0.120 | 0.055 | 0.070 | 0.704 |
| **Association between anterior overhang and anterior-TBR 2 years after surgery** | | | | | | | | |
| **Anterior-TBR** | 0.95±2.07  (-3.83 to 6.95) | 0.44±1.62  (-2.83 to 5.78)^3^ | -0.79±0.85  (-0.85 to -0.73) |  | 0.297 | 0.533 | 0.429 | 0.665 |

1. anatomically sized, 0mm - 1mm; mild over-hang, 1mm - 3mm; severe over-hang, ≥ 3mm.

2. One-Way ANOVA

3. Tukey post hoc test or Games-Howell post hoc test,

SD, standard deviation; TBR, tibial bone resorption.

**Table S2. Association between alignment and tibial bone resorption at 2 years after surgery.**

|  | **Aligned** | **Varus** | **Valgus** |  | **P^1^** | **P^2^**  **(varus VS aligned)** | **P^2^** | **P^2^**  **(varus VS valgus)** |
| --- | --- | --- | --- | --- | --- | --- | --- | --- |
|  | **Mean ±SD (range)** | | |  |  |  | **(valgus VS aligned)** |  |
| **Association between AP-TA and TBR 2 years after surgery** | | | |  |  | | | |
| **Medial-TBR** | 2.08±2.41  (-2.55 to 9.24) | 2.30±2.32  (-0.43 to 8.21) | 0.03±1.63  (-1.67 to 3.04) |  | 0.110 | 0.942 | 0.104 | 0.120 |
| **Lateral-TBR** | 1.06±2.63  (-3.14 to 9.37) | 1.25±2.17  (-1.60 to 6.67) | 0.29±1.96  (-2.48 to 3.45) |  | 0.730 | 0.961 | 0.753 | 0.714 |
| **Association between AP-TFA and TBR 2 years after surgery** | | | |  |  | | | |
| **Medial-TBR** | 1.64±2.18  (-2.55 to 9.24) | 2.67±2.40  (-1.44 to 8.85) | 2.26±4.03  (-1.67 to 8.95) |  | 0.121 | 0.104 | 0.815 | 0.921 |
| **Lateral-TBR** | 1.15±2.66  (-3.14 to 9.37) | 0.85±2.38  (-2.80 to 6.67) | 0.91±2.16  (-2.20 to 3.45) |  | 0.853 | 0.847 | 0.974 | 0.999 |
| **Association between L-TA and TBR 2 years after surgery** | | | |  |  | | | |
| **Anterior-TBR** | 0.93±1.96  (-5.98 to 3.62) | 0.30±1.52^3^  (-3.01 to 3.24)^3^ | |  | 0.407 |  |  |  |

1. For AP-TA and AP-TFA, One-Way ANOVA, for L-TA, student's t test

2. Tukey's post hoc test or Games-Howell post hoc test

3. Misaligned for L-TA

SD, standard deviation; AP-TA, anteroposterior tibial angle; TBR, tibial bone resorption; AP-FTA, anteroposterior tibial-femoral anatomical angle; L-TA, lateral tibial angle.

**Table S3. Association between** **alignment and PROMS (KSS total-post and WOMAC pain-post) at 2 years after surgery.**

|  | **Aligned** | **Varus** | **Valgus** |  | **P^1^** | **P^2^** | **P^2^** | **P^2^** |
| --- | --- | --- | --- | --- | --- | --- | --- | --- |
|  | **Mean ±SD (range)** | | |  |  | **(varus VS aligned)** | **(valgus VS aligned)** | **(varus VS valgus)** |
| **Association between AP-TA and PROMS 2 years after surgery** | | | |  |  | | | |
| **KSS total-post** | 177.26±13.09  (137 to 197) | 177.67±17.49  (127 to 196) | 182.33±16.99  (149 to 196) |  | 0.691 | 0.994 | 0.666 | 0.768 |
| **WOMAC pain-post** | 3.20±2.06  (0 to 8) | 2.53±1.96  (0 to 6) | 1.83±2.71  (0 to 7) |  | 0.182 | 0.483 | 0.267 | 0.766 |
| **Association between AP-TFA and PROMS 2 years after surgery** | | | |  |  | | | |
| **KSS total-post** | 177.01±13.97  (137 to 197) | 177.26±14.29  (127 to 196) | 186.17±7.73  (174 to 196) |  | 0.299 | 0.996 | 0.271 | 0.318 |
| **WOMAC pain-post** | 3.13±2.15  (0 to 8) | 3.15±2.02  (0 to 8) | 1.33±1.21  (0 to 3) |  | 0.122 | 0.999 | 0.108 | 0.123 |
| **Association between L-TA and PROMS 2 years after surgery** | | | |  |  | | | |
| **KSS total-post** | 177.27±14.05  (127 to 197) | 182.29±10.53^3^  (163 to 192)^3^ | |  | 0.358 |  |  |  |
| **WOMAC pain-post** | 3.04±2.11  (0 to 8) | 3.00±2.00^3^  (0 to 6)^3^ | |  | 0.962 |  |  |  |

1. For AP-TA and AP-TFA, One-Way ANOVA, for L-TA, student's t test

2. Tukey's post hoc test or Games-Howell post hoc test

3. Misaligned for L-TA

SD, standard deviation; AP-TA, anteroposterior tibial angle; PROMS, patient reported outcome measures KSS score, the Knee Society Score; WOMAC score, the Western Ontario and McMaster Universities Osteoarthritis Index score; AP-FTA, anteroposterior tibial-femoral anatomical angle; L-TA, lateral tibial angle.
